# An epidemiological study of urinary incontinence and its impact on quality of life among women aged 35 years and above in a rural area

**DOI:** 10.4103/0970-1591.70566

**Published:** 2010

**Authors:** Trupti N. Bodhare, Sameer Valsangkar, Samir D. Bele

**Affiliations:** Department of Community Medicine, Prathima Institute of Medical Sciences, Karimnagar, AP, India

**Keywords:** Quality of life, risk factors, urinary incontinence

## Abstract

**Background:**

There have been few community-based epidemiological studies on urinary incontinence (UI) evaluating the risk factors and impact on quality of life (QOL) in India.

**Objectives:**

This study was designed (1) to estimate age-specific prevalence and risk factors of UI among women aged 35 years and above in a rural area and (2) to analyze the impact of UI on the QOL of incontinent women.

**Design and Setting:**

A cross-sectional descriptive study was conducted.

**Materials and Methods:**

A semi-structured questionnaire assessing socio-demographic factors, severity and type of incontinence, and obstetrical and other risk factors along with impact on QOL was administered in two clusters (villages) in Karimnagar district through multistage cluster sampling.

**Results:**

In a sample of 552 women, 53 (10%) reported episodes of UI. The prevalence of UI showed significant association with increasing age (*P* < 0.01). Fifty-seven percent of the women had symptoms of stress incontinence, 23% of urge, and 20% mixed symptoms. Obstetrical factors associated with UI included high parity (*P* < 0.003), young age at first childbirth (*P* < 0.01), forceps delivery (*P* < 0.001), and prolonged labor (*P* < 0.001). Chronic constipation, chronic cough, and history of urinary tract infection were predictors of UI in regression analysis (Nagelkerke R ^2^= 0.7). Women with stress incontinence had the severest perceived impact on QOL on a five-point scale questionnaire, mean 24.87 (95% CI 21.26-28.47).

**Conclusion:**

One in 10 women reported episodes of UI with impaired QOL. The outcome is predicted both by obstetric and other risk factors.

## INTRODUCTION

Urinary incontinence (UI), defined by the International Continence Society as “the complaint of any involuntary leakage of urine”[1] is a common and distressing medical condition, severely affecting quality of life (QOL).[2] With prevalence ranging from 10% to 34%,[3,4] the condition is usually under reported as many women hesitate to seek help or report symptoms to medical practitioners due to the embarrassing and culturally sensitive nature of this condition.[5] Potential risk factors for UI include increasing age, increasing parity, vaginal deliveries, obesity, surgery, constipation, and chronic respiratory problems.[6,7] The problem leads to many women adopting detrimental changes in lifestyle to combat it and may even lead to feelings of shame and depression or even avoidance of social, work events, and sexual activities. Comorbid conditions such as urinary tract infections (UTIs), skin problems such as rashes, infections, and sores occur due to constantly wet skin. Economic burden due to increased costs and efforts for linen washing and healthcare adds to the consequences of this condition.[2] Little data exist on the prevalence, causative risk factors, and its impact on QOL in India. This study examined the age-specific prevalence, associated risk factors, and the impact of UI on QOL in a cross-sectional descriptive study in women aged 35 years and above. Women aged 35 years and above were selected as the prevalence rises in the cases of elderly women and completion of their obstetrical career ensures the removal of the confounding effects of obstetrical risk factors.

## MATERIALS AND METHODS

A cross-sectional study was conducted in two villages of Karimnagar district selected by multistage cluster sampling. A semi-structured questionnaire was designed which consisted of five parts: (1) Socio-demographic profile, (2) incontinence, its types and severity, (3) obstetric history, (4) risk factors for incontinence, and (5) impact of UI on QOL. One Assistant Professor and two female house surgeons previously trained regarding interviewing the respondents explained the questionnaire in lay language and recorded the responses. Pretesting and validation of the questionnaire were performed via a pilot study in 50 women. The questionnaire was administered in the two selected clusters in every household with women aged over 35 years. The purpose of the study was explained, and informed consent was obtained from all respondents. Privacy and confidentiality were ensured during the whole process. A total of 552 women consented and participated in the study.

Operational definitions for UI, stress, urge, and mixed incontinence were based on those provided by International Incontinence Society.[1] A woman was considered to have stress incontinence if there was a positive response to the question “Do you have episodes of involuntary leakage on efforts or exertion, or on sneezing or coughing?” and urge incontinence if there was a positive response to the question “Do you have episodes of involuntary leakage accompanied by or immediately preceded by urgency?” If a positive response was obtained for both questions, she was classified as a case of mixed incontinence.[7,8] Women with episodes of incontinence over a month were chosen for the present study. Women with acute UTIs, diabetes, neurological disorders, and similar conditions were excluded from the analysis.

Birth order greater than two was considered to be high parity; age of first delivery below 18 years was evaluated as a risk factor for outcome of UI. Among the mode of delivery, a woman was classified into the caesarean group if she had a history of exclusive caesarean delivery. If there was a history of at least one vaginal delivery or forceps delivery, she was grouped under vaginal delivery and forceps delivery, respectively. If the duration of any labor (first and second stages collectively) was more than 18 h, the women was considered to have had a prolonged labor. Birth weight of any child more than 3.5 kg was evaluated as a risk factor for UI.

We obtained history of chronic constipation, chronic cough, dilatation and curettage, any type of pelvic surgery, UTI, and pelvic infection. The above-mentioned factors were evaluated as predictors for outcome of UI.

Disabling symptoms affecting QOL were measured on five dimensions, namely activity limitation (domestic, occupational work, and travel over long distances), limitation of social interaction (social, religious, and leisure activities), limitation of sexual activities (sexual gratification, sexual activities, and fear of rejection by spouse), increased financial burden (medical care and laundry), and emotional upset and distress. The responses were graded on a five-point scale from 0 to 4 where zero corresponded to no effect at all and four was the maximal with QOL severely affected.

Data analysis was done using PASW (SPSS) software, version 18. The statistical measures obtained were means, confidence interval levels, Chi-square values, and logistic regression analysis values.

## RESULTS

The overall prevalence of UI among the study population was 53 (10%). The baseline characteristics of the sample respondents and age-specific prevalence of UI are described in Table 1. There was a significant association between increasing age and the outcome of UI. (χ^2^= 14.18, d.f. = 5, *P* < 0.01). Kuppuswamy Scale was used to evaluate the socio-economic status of the respondents and most belonged to the upper-lower (60%) or lower class (34%). There was no significant association between the socio-economic, marital status, and body mass index of the respondents and the outcome of UI. The severity and type of UI are described in Table 2. The most prevalent type of incontinence was stress incontinence (57%) followed by urge (23%) and mixed incontinence (20%). The median number of episodes of incontinence in the previous month for the sample respondents was ten episodes (10.46–17.16 95% CI). Thirty-six percent of the women reported a median of three (2.43–3.88) episodes per month, 32% reported a median of 10.5 (9.07–11.17) while 19% reported a median of 21.5 (18.47–23.72) episodes per month, and 13% of the women reported a median of 36.5 (33.85–40.89) episodes per month. The most severity with more than 30 episodes per month was observed in women suffering from stress incontinence. Table 3 describes the role of obstetrical factors in UI. Among the obstetrical factors, parity, age at first delivery, history of forceps delivery, and prolonged labor were significant for outcome of UI. Other risk factors playing a role in UI are enumerated in Table 4. A moderate positive correlation between the factors and outcome of UI was obtained (Nagelkerke *R*^2^ value = 0.7). The individual significant factors included chronic constipation (*P* < 0.001), chronic cough (*P* < 0.001), and UTI (*P* < 0.001). Table 5 describes the impact of UI on the QOL of women. Of a maximal obtainable score of 44, the mean score for the group of women with stress incontinence was the highest at 24.87 (21.26–28.47) followed by the group with mixed incontinence 18.18 (15.27–21.09) and lastly urge incontinence 15.91 (10.67–21.16).

**Table 1:** Baseline characteristics of the sample respondents

	Urinary incontinence absent	Urinary incontinence present
Age* (years)		
35–40	121	9
41–45	78	5
46–50	63	8
51–55	66	1
56–60	61	11
> 60	110	19
Marital status		
Married	404	42
Divorced	17	1
Widow	77	10
Unmarried	1	0
Socio-economic status		
Upper middle	12	2
Lower middle	15	4
Upper lower	287	46
Lower	185	1
BMI		
<18	57	3
18–25	371	48
>25	71	2
Total	499	53

* Age is significant for outcome of urinary incontinence, *P* < 0.01.

**Table 2:** Type and severity of urinary incontinence

Frequency of episodes	Stress incontinence	Urge incontinence	Mixed incontinence	Total	Mean ± SD (95% CI)	Median
Few episodes per month	8	6	5	19	3.16 ±1.5 (2.43–3.88)	3
Few episodes per fortnight	10	3	4	17	10.13 ± 1.96 (9.07–11.17)	10.5
Few episodes per week to one episode per day	5	3	2	10	21.1 ± 3.67 (18.47–23.72)	21.5
More than one episode per day	7	0	0	7	37.38 ± 4.2 (33.85–40.89)	36.5
Total (n)	30	12	11	53	13.81 ± 12.16 (10.46–17.16)	10

**Table 3:** Obstetrical factors playing a role in urinary incontinence

	Urinary incontinence absent	Urinary incontinence present	*P* value
Parity			
Nulliparous	24	1	0.004
1–2 children	273	18	
>2 children	63	8	
Age at first delivery			
<18 years	191	32	0.01
18–22 years	156	11	
>22 years	128	9	
NA	24	1	
Vaginal delivery or caesarean			
Vaginal	448	42	0.001
Caesarean	27	10	
NA	24	1	
Forceps delivery			
No	473	48	0.001
Yes	2	4	
NA	24	1	
Prolonged labor			
No	463	37	0.001
Yes	12	15	
NA	24	1	
Maximal birth weight			
<2.5 kg	128	10	0.13
2.5–3.5 kg	276	29	
>3.5 kg	71	13	
NA	24	1	
Total	499	53	

**Table 4:** Other risk factors playing a role in urinary incontinence*

	Urinary incontinence absent	Urinary incontinence present	*P* value
Chronic constipation			
No	452	5	0.000
Yes	47	48	
Chronic cough			
No	439	37	0.01
Yes	60	16	
Dilatation and curettage			
No	421	48	0.291
Yes	78	5	
History of pelvic surgery			
No	370	39	0.232
Yes	129	14	
History of urinary tract infection			
No	415	28	0.001
Yes	84	25	
History of pelvic infection			
No	419	39	0.722
Yes	80	14	

* Nagelkerke R^2^ = 0.7

**Table 5:** Impact of urinary incontinence on the quality of life

Disabling symptoms	Stress incontinence Mean (95% CI)	Urge incontinence Mean (95% CI)	Mixed incontinence Mean (95% CI)
Activity limitation	6.87 (5.85–7.88)	5.75 (4.48–7.02)	5.09 (4.22–5.96)
Social interaction limitation	6.87 (5.74–7.99)	3.75 (2.1–5.4)	5.73 (4.64–6.81)
Sexual activity limitation	5.57 (4.69–6.45)	3.58 (2.3–4.9)	3.27 (2.67–3.88)
Financial burden increased	3 (2.67–3.33)	1.25 (0.7–1.8)	2.18 (1.68–2.69)
Emotional upset and distress	2.57 (2.17–2.97)	1.58 (1.01–2.16)	1.91 (1.55–2.27)
Mean	24.87 (21.26–28.47)	15.91 (10.67–21.16)	18.18 (15.27–21.09)

## DISCUSSION

The overall prevalence of UI in this study was 10%. The distribution of the types of incontinence was as follows: 57% women had stress incontinence, 23% had urge, and 20% had mixed type. These findings were similar to the study conducted by Kumari *et al*., who reported the overall prevalence of UI as 12%, among whom 46% had stress incontinence, 26% had urge, and 28% had mixed type.[3] It is lesser than the global prevalence of UI which may be due to variations in definitions used, age groups and populations studied.[4]

Urinary incontinence is a significant health problem in the community, leads to embarrassment, curtailment of daily, social and sexual activities and is a considerable economic burden on the individual as well as the healthcare system.[2,9] Identification of risk factors of UI and altering them can reduce this burden. Prevalence of UI increases with advancing age, and the etiology of this association is unclear. This is partly explained by progressive loss of muscle tone, decreased contractility, changes in the hormonal stimulation, and repeated injuries during parturition.[9,10] In this study, the prevalence of UI increased with age. Childbearing is an established risk factor for UI; the labor and delivery process may cause pelvic floor dysfunction as a result of nerve damage, muscular damage, and direct tissue stretching and disruption.[6,9,10] Sixty-four percent of the incontinent women had borne more than two children in their obstetrical career in the current study. Among the modes of delivery, the women who had undergone a caesarean section had a higher proportion of outcome of UI. This may be explained by the fact that emergency caesarean section may not be protective for UI[11] which was the norm in our sample. There is a significant association between the outcome of UI and history of forceps delivery. Eighty-three percent of the women who had incontinence also had a age of first childbirth of 22 years or lesser which is similar to the study conducted by Fritel *et al*.[11] Immaturity of the reproductive system and child bearing below the age of 18 years may predispose a woman for UI. Further, in India, marriage and childbearing are traditionally at younger ages and in our study, 62% of the incontinent women had their first childbirth at an age less than 18 years. A study by Morley *et al*.[12] concluded that prolonged labor causes collagenous changes in the pelvic floor leading to UI. In this study, we found a highly significant association of UI with prolonged duration of labor.

The association between constipation, chronic cough, and UI can be explained by increased abdominal pressure.[13] Both chronic cough and constipation were significant predictors for UI in the regression analysis in our study. There was an association between history of UTI and UI similar to that found in studies conducted by van Gerwen *et al*.[13] and Olsson *et al*.[14]

There was a clear-cut relationship between measures of severity and measures of distress, impaired QOL with 17 women reporting more than one episode per day to one episode per week and reporting high mean scores on the QOL scale questionnaire. Stress incontinence was the commonest type of incontinence observed and also had the most severity and greatest impact on QOL. The impact was equitable over all dimensions measured for QOL, namely activity limitation, social interaction limitation, sexual activity limitation, increased financial burden, and emotional upset and distress.

## LIMITATIONS

In this study, the data included were obtained solely on verbal response, recall bias for some obstetrical factors may have been present, the perception of QOL may have been subjective, and intervention could not be taken up due to limited resources and time constraint.

## CONCLUSION

Almost 1 in 10 women reported suffering from episodes of UI. Simple epidemiological tools such as a questionnaire can unveil the incontinence subjectively. The outcome is predicted both by obstetric and other risk factors. Further study is required to delineate the individual factors playing a role in stress versus urge incontinence. There is a significant impact of UI on QOL. Mitigating the effect of UI and improving their QOL in women will require further understanding of their coping skills and their perceptions of themselves.

## Appendix

### An epidemiological study of urinary incontinence and its impact on quality of life among women aged 35 years and above in a rural area: A questionnaire

**Part A: General demographic information**

1. **Name:** ______________________________________________
2. **Age:** ______
3. **Marital status:** □Married □Divorced □Widow □Unmarried
4. **Education:** □Professional □Graduate or post graduate □Post high school diploma □High school □Middle school □Primary school □Illiterate
5. **Occupation:** □Professional □Semi-Professional □Clerical, Shop owner, Farmer □Skilled worker □Semi-skilled worker □Unskilled worker □Unemployed
6. **Total family monthly income:** ______________________________________
7. **Weight:** ________________________
8. **Height:** _______________________

**Part B: Specific information**

1. In the past one month have you leaked urine involuntarily? □Yes □No
2. If yes, how many times in the last month has this happened? _____________________
3. Since how long have you been experiencing this involuntary leakage? _____________
4. Do you have episodes of involuntary leakage on efforts or exertion, or on sneezing or coughing? □Yes □No
5. Do you have episodes of involuntary leakage accompanied by or immediately preceded by urgency? □Yes □No
6. Do you have to rush to the toilet to urinate? □Yes □No
7. How often do you usually need to urinate in a day? ___________________________
8. Do you get up at night to void urine? □Yes □No If yes, how often? ____________
9. Does this frequency of voiding affect your activities? □Yes □No
10. Did you at any time seek medical advice for your problem of involuntary loss of urine? □Yes □No
11. Do you currently take any medications? □Yes □No If yes, details ______________
12. Do you suffer from the following conditions? □Diabetes □Hypertension □Heart disease □Stroke □Neurological conditions □Psychiatric conditions □UTI

**Part C: Obstetric history**

1. **Age at marriage:** ______________
2. **Age at menarche:**_______________
3. **Menopause attained** □Yes □No
4. Did you learn perineal exercises during your antenatal or postnatal period? □Yes □No

**Table:**

Pregnancy number*	Age at delivery	Mode of delivery (details)	Duration of labor	Weight of the baby	ANC received	PNC received	Immediate postnatal complications

* Enter additional births in a separate sheet

**Part D: Other factors**

1. Have you suffered from constipation? □Yes □No If yes, from how long? ________
2. Have you suffered from persistent cough lasting for more than eight weeks? □Yes □No
  If yes, from how long? ___________________________________________________
3. Have you undergone any type of pelvic surgery in the past? □Yes □No
4. **History of UTI in the past year** □Yes □No. If yes, number of episodes ____________________ and treatment obtained ________________________
5. **History of PID** □Yes □No. If yes, treatment obtained __________________
6. **History of dilation and curettage** □Yes □No

**Part E: Quality of life**

**Table:**

To what extent do you feel that this condition	Never	Little	Somewhat	Much	A great deal
Prevented you from doing domestic activity?					
Prevented you from doing occupational work?					
Prevented you from travelling long distances?					
Prevented you from interacting with other people?					
Prevented you from attending religious ceremonies and other functions?					
Prevented leisure activities?					
Caused you to reduce sexual activity?					
Reduced your sexual gratifi cation?					
Invoked in you a fear of rejection by spouse?					
Caused additional financial burden through medical care or laundry?					
Invoked negative feelings in you such as blue mood, despair, anxiety, depression?					
